# Identifiability Limits of Forward-Osmosis Membrane Transport from Water and Reverse-Solute Flux Observations

**DOI:** 10.3390/membranes16100339

**Published:** 2026-10-13

**Authors:** Maurizio Viviani, Nicola Bragazzi, Simonetta Papa, Luca Giacomelli, Roberto Eggenhöffner

**Affiliations:** 1 Robotics.it LLC, 9649 West Olympic Blvd, Suite 7, Beverly Hills, CA 90212, USA; digimatronics@gmail.com; 2 Laboratory for Industrial and Applied Mathematics (LIAM), Department of Mathematics and Statistics, York University, Toronto, ON M3J 1P3, Canada; 3 Polistudium SrL, Via Solferino 7, 20121 Milan, Italy

**Keywords:** forward osmosis, admissible inference, non-identifiability, observable rank, Fisher information

## Abstract

Forward osmosis (FO) membrane performance is commonly described through water flux *J_w_*, reverse-solute flux *J_s_*, and the specific reverse-solute ratio *R_J_* = *J_s_*/*J_w_*. These observables provide a low-dimensional projection of a coupled transport system where selective-layer permeability, support resistance, concentration polarization, and defect-mediated leakage interact, producing non-unique links between membrane organization and measured performance. The objective of this study is to determine to what extent the commonly reported FO observables *J_w_*, *J_s_*, and *R_J_* can constrain the underlying transport mechanisms, and to quantify the residual ambiguity when different combinations of selective-layer permeability, support resistance, concentration polarization, and defect-mediated leakage remain compatible with the same measured response. To address this objective, the study develops an admissible-inference framework in the experimentally accessible (*J_w_*, *R_J_*) plane, where published FO measurements are analyzed through observable-space mapping, concentration-polarization amplification, covariance-rank analysis, Fisher/SVD sensitivity diagnostics, and admissible-volume mapping. The resulting framework determines which transport interpretations remain compatible with the observations and which mechanistic directions require additional independent measurements to be resolved.

## 1. Introduction

Forward osmosis (FO) performance emerges from coupled water permeation, reverse-solute transport, concentration polarization, and membrane heterogeneity [1,2,3], so water flux alone is an incomplete measure of membrane performance. The specific reverse-solute ratio, *R_J_* provides a compact measure of this balance by expressing reverse-solute leakage relative to useful water transport [4,5].

This ratio is useful for benchmarking, but it does not identify the physical origin of the measured response. Similar *R_J_* values can result from different combinations of selective-layer permeability, support resistance, internal or external concentration polarization, and defect-mediated leakage [5,6]. This ambiguity is particularly relevant for heterogeneous and modified FO membranes because surface functionalization, filler loading, support morphology, and local defects can affect several transport contributions simultaneously [7].

Conventional FO interpretation often relies on the comparison of measured water flux, reverse-solute flux, and specific reverse-solute behavior across membranes, operating conditions, or draw-solution compositions [8,9,10,11]. These comparisons are indispensable for experimental benchmarking, but they leave unresolved whether an observed change in performance originates from improved intrinsic selectivity, reduced concentration polarization, altered support transport, or leakage pathways unresolved by macroscopic flux measurements alone. Representative FO studies spanning the experimental basis used in the present analysis and complementary physical characterization are summarized in Table 1. Together, they show that water and reverse-solute fluxes are widely reported for performance comparison, whereas independent measurements capable of constraining permeability, support/polarization behavior, or selective-layer structure are much less consistently available. This asymmetry provides the experimental basis for the identifiability problem addressed here: membrane performance can be quantified directly from the flux observables, while its physical origin may remain non-unique unless an additional independent observable constrains the unresolved transport direction.

The limitation is structural: a limited set of macroscopic observables is used to infer a transport architecture that may contain several coupled and partially compensating mechanisms. It can be expressed as the distinction between deterministic reconstruction and admissible inference [12,13]. Deterministic reconstruction requires the observables to identify a unique membrane state or a narrowly bounded parameter set. Comparative FO datasets rarely satisfy this condition because several interacting mechanisms are inferred from a small number of macroscopic measurements. Admissible inference instead determines which transport states remain compatible with the observations after physically plausible bounds and model constraints have been imposed. The information limit is also explicit in the observable definitions. Because *R_J_* = *J_s_*/*J_w_*, the nominal triplet (*J_w_*, *J_s_*, *R_J_*) contains at most two independent scalar quantities. Improved measurement precision can narrow the uncertainty associated with these quantities, but it cannot create an additional independent observable direction. Unique structural interpretation therefore requires measurements that are not algebraically derived from *J_w_* and *J_s_*. Representative studies spanning flux-based performance assessment and complementary transport or structural characterization are compared in Table 1.

**Table 1:** State-of-the-art comparison of representative FO studies relevant to the present identifiability analysis.

Reference	FO Membrane/System	Feed/Draw or Operating Context	Transport/Characterization Focus	Water Permeation	Reverse-Solute Transport	Support Resistance/CP/Complementary Physical Information
**Studies contributing to the harmonized observable dataset**						
Phillip et al. [4].	Commercial HTI-CTA	NaCl draw solution, 1–4 M	Draw-concentration dependence of FO transport	*J_w_* ≈ 5–20 LMH	*R_J_* ≈ 0.24–0.26 g L^−1^; reverse-solute transport reported in the source dataset	Flux-based characterization; support/CP contributions are not independently resolved by the observable pair
Rufuss et al. [14].	Aquaporin HFFO.6 hollow-fiber membrane	NaCl draw; concentration, temperature, and flow varied	Operating-condition dependence of coupled FO transport	*J_w_* ≈ 6.6–33 LMH	*R_J_* ≈ 0.22–0.80 g L^−1^; *J_s_* reported	Operating perturbations expose changes in the coupled response, but do not independently isolate support/CP contributions
Nasr et al. [15].	PES/GO mixed-matrix membranes; HTI-CTA reference	NaCl draw, 0.65–2 M	Membrane modification and draw-concentration response	SG3: *J_w_* ≈ 105–127 LMH; CTA ≈ 7.2–15 LMH	SG3: *R_J_* ≈ 2.6 × 10^−4^–1.9 × 10^−2^ g L^−1^; CTA ≈ 0.28–0.35 g L^−1^	Strongly separated observable responses; flux data alone do not uniquely partition permeability, support/CP, and leakage contributions
Idris et al. [16].	TFN-GO membrane series, 0–1.0 wt% GO	1 M NaCl draw; GO loading varied	Selective-layer/material modification	*J_w_* reported across the GO-loading series	*J_s_* reported; *R_J_* obtained from paired flux observations	Non-monotonic material-series response illustrates that compositional modification can alter several transport contributions simultaneously
**Independent experimental case studies used to examine interpretations unresolved by the conventional flux observables**						
Kallem et al. [17].	TFC-PES and TFC-PES/PDA@TiO_2_	Oily-wastewater treatment; membrane modification	FO transport combined with independent permeability characterization	FO *J_w_*: 12.5 → 34.3 LMH; water permeability: 1.62 → 3.19	*J_s_*: 6.1 → 9.2 g m^−2^ h^−1^; *R_J_*: 0.488 → 0.268 g L^−1^; salt permeability: 1.11 → 1.23	Independent water- and salt-permeability measurements provide information unavailable from the FO flux pair alone
He et al. [18].	Polyamide selective layers prepared under different interfacial-polymerization conditions	Selective-layer structure varied	FO transport combined with morphology/structure characterization	FO water transport reported	Reverse-solute transport reported; lower leakage associated with the denser selective layer	Morphological evidence on layer continuity and defects provides an independent constraint on leakage-related interpretation
Bai and Kruczek [19].	Same commercial osmotic membrane in AL-FS and AL-DS orientations	Orientation and draw concentration varied	Orientation-dependent water and salt transport dynamics	Water-flux response depends on membrane orientation	Salt-transport time lag ≈ 4.3–4.6 min in AL-FS and ≈ 7–20 min in AL-DS	Orientation changes support/ICP conditions without changing membrane chemistry; time-lag data add an independent constraint on support/polarization interpretation

Across these studies, water and reverse-solute fluxes are broadly available for performance comparison, whereas measurements that independently constrain permeability, support/polarization behavior, or selective-layer structure are less consistently reported. This asymmetry is the experimental basis for the identifiability question addressed here: performance differences can be measured directly, but their physical origin may remain non-unique unless an additional independent observable constrains the unresolved transport direction.

The objective of this study is to determine to what extent the commonly reported FO observables *J_w_*, *J_s_*, and *R_J_* can uniquely constrain the underlying transport mechanisms, and to quantify the residual ambiguity when different combinations of selective-layer permeability, support resistance, concentration polarization, and defect-mediated leakage remain compatible with the same measured response.

To address this objective, published FO measurements are organized in the experimentally accessible (*J_w_*, *R_J_*) plane and analyzed using concentration-polarization amplification, continuous transport weights, covariance-rank analysis, Fisher/SVD sensitivity diagnostics, and admissible-volume mapping. These complementary analyses determine which transport interpretations remain compatible with the observations and which mechanistic directions require additional independent measurements to be resolved.

The framework combines three complementary components. Classical FO transport relations provide the physical basis for water permeation, reverse-solute leakage, support resistance, and concentration polarization. Observable-space analysis then organizes published measurements without assigning a unique membrane structure or transport mechanism to each experimental point, while rank and sensitivity diagnostics determine how many transport-support directions are independently constrained and quantify the residual admissible region. The central issue addressed by this framework is therefore not a limiting FO transport performance, but the identifiability of the mechanisms underlying the observed performance.

This objective differs from the coherence-loss analysis reported previously [20]. In that study, *R_J_* was used primarily to characterize transport deterioration, whereas here *R_J_*, together with *J_w_*, is used to determine which mechanistic interpretations remain distinguishable from the available measurements. The present analysis therefore does not seek to establish a new membrane-performance ranking or to demonstrate higher transport efficiency than previous FO systems. Its methodological contribution is to quantify the extent to which commonly reported FO observables constrain competing physical interpretations and to identify when additional independent information is required to reduce the remaining ambiguity.

To examine this inference logic experimentally, three literature case studies were additionally selected to represent distinct sources of ambiguity associated with membrane permeability, support and polarization effects, and selective-layer structure. These cases are treated separately from the primary dataset and are not used to recalibrate the reduced model. Instead, they provide experimental examples of how complementary measurements can discriminate among transport interpretations that remain unresolved when only *J_w_*, *J_s_*, and *R_J_* are available.

## 2. Materials and Methods

### 2.1. Observable Transport Quantities

The proposed framework is predicated on experimentally measurable first-order transport quantities. The volumetric water flux is represented by *J_w_*, the reverse-solute flux by *J_s_*, and the specific reverse-solute ratio, following established FO usage [4,20], by:

*R_J_* = *J_s_*/*J_w_* (1)

Throughout this work, *J_w_* represents water productivity, *J_s_* represents reverse-solute leakage, and *R_J_* expresses leakage per unit water flux. Low *R_J_* values indicate that reverse-solute transport is small relative to useful water transport, whereas high values indicate a progressively larger leakage penalty.

The use of *R_J_* follows the preceding coherence-loss framework, but its role is extended here. Equation (1) follows the standard FO definition of specific reverse-solute transport used in the source literature [4,20]. The previous analysis used *R_J_* mainly as a diagnostic descriptor; the present analysis uses it as one coordinate of the observable space in which compatible transport states are identified. A given *R_J_* value can still arise from different physical mechanisms and therefore cannot serve as a structural coordinate by itself.

The observed transport data are denoted as *D* = (*J_w_*, *J_s_*, *R_J_*). This representation reflects the information commonly available in comparative FO studies, which generally report fluxes without enough independent structural measurements to identify a complete inverse model. The following analysis therefore begins from the reported transport observables and introduces structural descriptors only within explicitly defined reduced models.

### 2.2. Observable Degeneracy and Admissible Inference

The analysis tests whether the available FO observables admit more than one underlying transport-support state. Let *θ* ∈ *Θ* denote a vector containing effective transport-support descriptors, such as selective-layer permeability, support resistance, defect fraction, or concentration-polarization coupling strength. Let ℳ(*θ*) denote the forward map from these underlying descriptors to measurable observables. In a fully identifiable problem, the inverse map from D to θ would be unique or narrowly constrained. When the forward map is many-to-one within the adopted parameter domain, the admissible set 𝒜(*D*) contains multiple transport-support configurations. For the present analysis, the admissible set associated with a given observation *D* is defined as

𝒜(*D*) = {*θ* ∈ Θ:ℳ(*θ*) ≈ *D*} (2)

where the symbol ≈ denotes compatibility within experimental variability and within the resolution of the simplified transport representation [12,13]. The set 𝒜(*D*) is not interpreted as a unique reconstructed membrane structure, but as a compatibility set containing transport-support states that cannot be excluded by the available observables. Its size and shape depend not only on membrane heterogeneity and concentration-polarization amplification, but also on the effective rank of the observable representation itself. Because *R_J_* is algebraically determined by *J_w_* and *J_s_*, the standard observable triplet is rank-limited unless additional independent measurements are introduced. Multiple structurally distinct configurations may therefore remain admissible even when observational uncertainty is narrow.

The size and geometry of 𝒜(*D*) indicate how strongly the observations restrict the reduced transport-support space. A narrow region corresponds to stronger restriction, whereas a broad or elongated region indicates directions along which substantially different states remain compatible with the data. Overlap between regions associated with different transport regimes indicates that the measured response does not distinguish uniquely among the corresponding mechanisms.

### 2.3. Effective Concentration-Polarization Amplification

The datasets were selected to sample qualitatively different regions of the (*J_w_*, *R_J_*) plane, including selective, transitional, leakage-dominated, and non-monotonic responses; they were not intended as an exhaustive survey of the FO literature. The algebraic rank limit derived in Section 2.7 applies to every dataset constructed from the same triplet (*J_w_*, *J_s_*, *R_J_*). The numerical extent and distribution of the admissible regions, however, remain specific to the selected datasets, uncertainty assumptions, reduced forward model, and parameter domain.

In the experimental dataset, the leakage ratio is represented by the measured quantity *R_J_*. Based on the established physical description of concentration-polarization effects on FO flux behavior [2], we introduce the following reduced phenomenological relation to represent CP-induced amplification of the leakage response:

*R_CP_* = *R*_0_*exp*(*J_w_*/*k_eff_*) (3)

where *k*_eff_ is an effective phenomenological coupling scale. Within Equation (3), lower *k*_eff_ values produce a stronger increase in *R*_CP_ with *J_w_*, whereas higher values produce weaker amplification. The parameter summarizes the combined influence of concentration polarization, support-related transport resistance, and flux-dependent leakage; it is not treated as an independently measured membrane constant. Varying *k_eff_* therefore provides a systematic way to assess how polarization uncertainty expands or contracts the admissible observable domains.

Equation (3) retains the coupling between water flux and apparent leakage using a single effective scale. It is used to examine observable displacement under different amplification strengths, not to estimate a unique intrinsic membrane parameter.

The physical motivation for Equation (3) is the established coupling between flux and concentration polarization in FO [2,5]; the single-scale exponential form used here is a phenomenological reduction.

### 2.4. Soft Admissibility Weights for Transport Regimes

Transport regimes are characterized by soft admissibility weights instead of rigid class labels. Three distinct regimes are identified: stable selective transport, transitional transport, and leakage-dominated transport. Their corresponding occupancy weights are denoted by *P_S_*(*R_CP_*), *P_T_*(*R_CP_*), and *P_L_*(*R_CP_*), respectively.

Stable-transport occupancy is assigned high weight when *R_CP_* falls below *R_S_*; leakage-dominated occupancy increases when *R_CP_* exceeds an upper characteristic threshold. For the present admissibility analysis, the transition between these limits is represented continuously using a standard logistic functional form [21]. In the present framework, this form is used to avoid artificial discontinuities associated with a sharp threshold in an observable coordinate measured with finite uncertainty; it is not intended to represent an intrinsic sigmoidal transport law:

(4) $$P_{S}(R_{\mathrm{CP}})={\left[1+\exp\left((R_{\mathrm{CP}}-R_{S})/\sigma_{S}\right)\right]}^{-1}$$

(5) $$P_{L}(R_{\mathrm{CP}})={\left[1+\exp\left(-(R_{\mathrm{CP}}-R_{L})/\sigma_{L}\right)\right]}^{-1}$$

where *σ_S_* and *σ_L_* indicate the degree of smoothness during the transition. The transitional admissibility weight is then represented here as the residual overlap:

*P_T_*(*R_CP_*) = max{0, 1 − *P_S_*(*R_CP_*) − *P_L_*(*R_CP_*)} (6)

followed by normalization when necessary so that *P_S_* + *P_T_* + *P_L_* = 1. In the illustrative implementation, the characteristic regime thresholds were set at *R_S_* = 0.05 g L^−1^ and *R_L_* = 0.40 g L^−1^. The smoothing widths *σ_S_* and *σ_L_* were chosen only to avoid artificial discontinuities and do not represent independently fitted physical parameters.

These functions are continuous compatibility weights, not Bayesian posterior probabilities, because no likelihood or prior distribution is specified. Their purpose is to replace discontinuous regime assignments with gradual transitions over the experimentally uncertain *R*_CP_ coordinate. The values quantify compatibility with the selective, transitional, and leakage-dominated regimes defined by the adopted thresholds.

Equations (4)–(6) are mathematical compatibility definitions introduced in the present work; they are not adopted from a prior FO transport law.

### 2.5. Construction of the Structural-Support Compatibility Landscape

The relation between the observables and the underlying transport descriptors is examined in a reduced (*A*, *f_d_*) plane. The coordinate *A* represents effective productive permeability, whereas *f_d_* represents defect-mediated or leakage-prone transport. These coordinates do not reproduce the complete membrane architecture; they provide a reduced representation of the compensation between productive permeability and loss of selectivity.

A compatibility landscape *W*(*A f_d_*∣*D*) identifies the regions of the reduced (*A*, *f_d_*) plane that remain consistent with the observed transport data *D*. Each parameter pair (*A*, *f_d_*) is assigned a weight between 0 and 1 according to the distance between its predicted (*J_w_*, *R_J_*) response and the observed response, scaled by the corresponding uncertainty widths. High values identify transport-support states compatible with the observation, whereas low values identify states that fall outside the adopted observational uncertainty envelope. From the inverse-problem/compatibility background [12,13] and within the present reduced inference framework, the compatibility weight is defined as:

(7) $$W(A,f_{d}|D)=\exp\left[-\frac{1}{2}{\left(\frac{J_{w}^{\mathrm{pred}}(A,f_{d})-J_{w}^{\mathrm{obs}}}{\sigma_{J_{w}}}\right)}^{2}-\frac{1}{2}{\left(\frac{R_{J}(A,f_{d})-R_{\mathrm{CP}}}{\sigma_{R_{J}}}\right)}^{2}\right]$$

The corresponding normalized compatibility field is defined as:

(8) $$\tilde{W}(A,f_{d}|D)=W(A,f_{d}|D)/\max_{A,f_{d}}W(A,f_{d}|D).$$

Here $\sigma_{J_{w}}$ and $\sigma_{R_{J}}$ are declared compatibility widths assigned to the two observable coordinates. They define the resolution of the comparison and are not presented as independently estimated experimental standard deviations. The forward-map calibration, numerical values of the widths, parameter bounds, grid, aggregation rule, and sensitivity analyses are reported in Sections S2.2–S2.5 of the Supplementary Materials. Within the present analysis, $\tilde{W}$ is a compatibility score rather than a likelihood or posterior probability and Equations (7) and (8) are definitions within the present compatibility construction.

### 2.6. Data Organization and Calculation Workflow

The calculation workflow proceeds through five coordinated stages. The workflow is structured to move progressively from experimentally reported observables to the identification of compatible transport states and, finally, to the assessment of which mechanistic directions remain unresolved by the available measurements. First, the published *J_w_* and *J_s_* values are converted to common units, and *R_J_* is calculated from Equation (1) when it is not reported directly. These quantities define the harmonized observable dataset *D* = (*J_w_*, *J_s_*, *R_J_*). Second, the observations are projected onto the (*J_w_*, *R_J_*) plane and examined for clustering, overlap, and non-monotonic trajectories. This step establishes the observable-space organization without assigning a unique structural interpretation to individual measurements.

Third, the measured leakage ratio is propagated through the reduced concentration-polarization relation of Equation (3). Representative values of *R*_0_ and *k*_eff_ are used to examine how flux-dependent amplification displaces an otherwise comparable transport response within observable space. Fourth, the logistic functions of Equations (4)–(6) are evaluated to obtain the continuous selective, transitional, and leakage-dominated weights *P_S_*, *P_T_*, and *P_L_*. These weights describe gradual regime compatibility and are calculated independently of the structural-support map.

Fifth, the reduced forward model maps each (*A*, *f_d_*) pair to a predicted (*J_w_*, *R_J_*) response. Equations (7) and (8) compare this prediction with the observed response and generate the normalized compatibility landscape $\tilde{W}(Af_{d}|D$). The admissible-volume fraction is then calculated by applying the fixed threshold *W*_thr_ to this field. Finally, covariance-rank and Fisher/SVD diagnostics are used to distinguish the independent observable directions from those that remain algebraically dependent or numerically null.

All stages use the same harmonized observable dataset but address different questions: observable projection describes the measured performance space; Equation (3) examines polarization-related displacement; Equations (4)–(6) quantify regime compatibility; Equations (7) and (8) identify compatible reduced transport-support states; and the rank-aware analysis evaluates the independent information carried by the observable representation. Table 2 summarizes the measured quantities, derived coordinates, effective parameters, compatibility functions, and diagnostic measures used in this workflow.

### 2.7. Observable-Rank Consistency and Admissible-Volume Fraction

A rank-aware consistency analysis was applied to the 26 observations in the harmonized dataset. The covariance audit used log *J_w_*, log *J_s_*, and log *R_J_*, with *R_J_* recomputed from the same *J_w_* and *J_s_* values for this audit so that the defining algebraic relation was retained at machine precision. The local sensitivity diagnostic was evaluated from the uncertainty-scaled Jacobian of the reduced two-parameter forward map. Complete construction and numerical settings are given in Section S2.2 of the Supplementary Materials.

For each observation, the admissible-volume fraction *f*_adm_ was calculated as the fraction of the declared (*A*, *f_d_*) grid satisfying |*W*| ≥ *W_thr_*. The same threshold, *W_thr_* = 0.22, was applied throughout the reference analysis. For the Gaussian compatibility score, this threshold corresponds to a combined standardized distance of approximately 1.74. System-level values are medians over the observations assigned to each membrane system. Threshold, width, and grid sensitivity are reported in the Supplementary Materials.

The observable covariance matrix was calculated in logarithmic coordinates from the harmonized dataset, and its normalized eigenvalues were used to determine the effective observable rank. The local sensitivity analysis was based on the Jacobian matrix of the reduced forward model, following standard identifiability and parameter-sensitivity analysis [22,23].

$$J_{\phi }=\frac{\partial (J_{w}^{\mathrm{pred}},R_{J}^{\mathrm{pred}})}{\partial (A,f_{d})}$$

The corresponding local Fisher information matrix was defined as

$$F(A,f_{d})=J_{\phi }^{T}J_{\phi }$$

The eigenvalues of *F*, equivalently the squared singular values of *J_ϕ_*, quantify the local sensitivity of the predicted observables to displacements in the reduced parameter space. The five-entry Fisher/SVD spectrum was constructed using the extended diagnostic representation described in Section S2.2 of the Supplementary Materials. Its first two entries identify the informative directions supported by the reduced mapping, whereas the remaining entries represent the algebraic and numerical rank collapse within the extended representation.

Threshold sensitivity was evaluated over *W*_thr_ = 0.10–0.40, and grid convergence was tested at 150 × 150, 300 × 300, and 500 × 500 resolution, as reported in Sections S2.4 and S2.5. Larger *f*_adm_ values indicate that a greater fraction of the declared reduced domain remains compatible with the observation; smaller values indicate stronger restriction of that domain. The quantity nevertheless depends on the observation, reduced forward model, uncertainty widths, threshold, grid, and parameter bounds and should not be interpreted as an intrinsic membrane property [12,13].

### 2.8. Case Studies: Disambiguation by Complementary Measurements

Three published FO studies were examined separately from the 26-observation harmonized dataset because they combine FO transport measurements with complementary physical information addressing distinct sources of mechanistic ambiguity. The selected cases provide independent characterization related to membrane transport properties, orientation-dependent support/polarization effects, and selective-layer structure, respectively [17,18,19].

The cases were not pooled with the primary dataset and were not used to refit or recalibrate the reduced model. They were examined separately to compare the interpretation supported by FO flux observables with that enabled by complementary physical characterization.

## 3. Results

### 3.1. Observable-Space Organization of FO Transport

**Figure 1:**
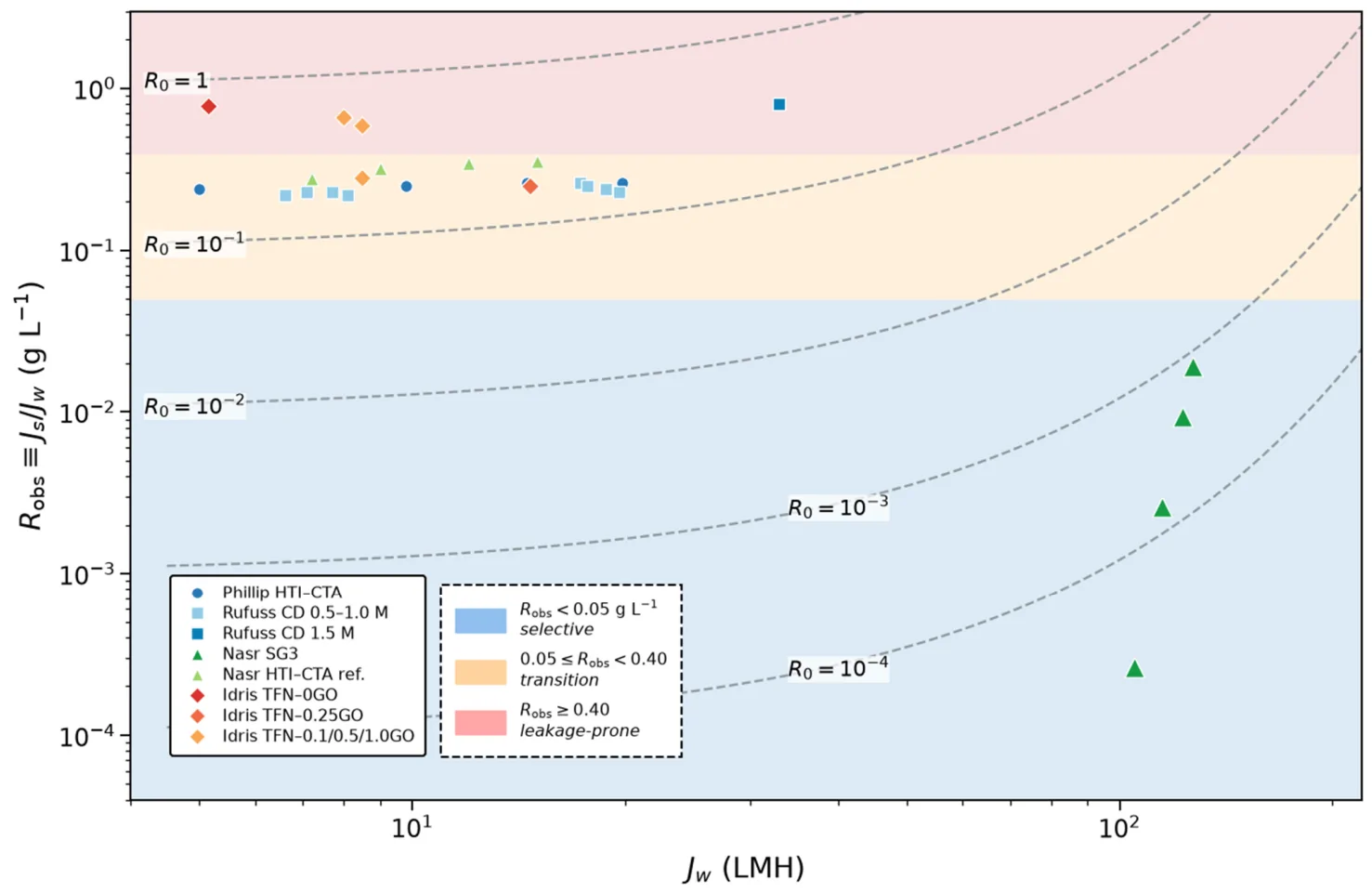
Observable-space organization of FO transport states. The 26 observations in Table 3 are projected into the (*J_w_*,*R_obs_*) plane, where *R_obs_* = *J_s_*/*J_w_*. Shaded bands show the declared selective, transitional, and leakage-prone ranges. Structurally distinct systems occupy neighboring observable regions, showing that these coordinates do not provide unique structural fingerprints.

**Figure 2:**
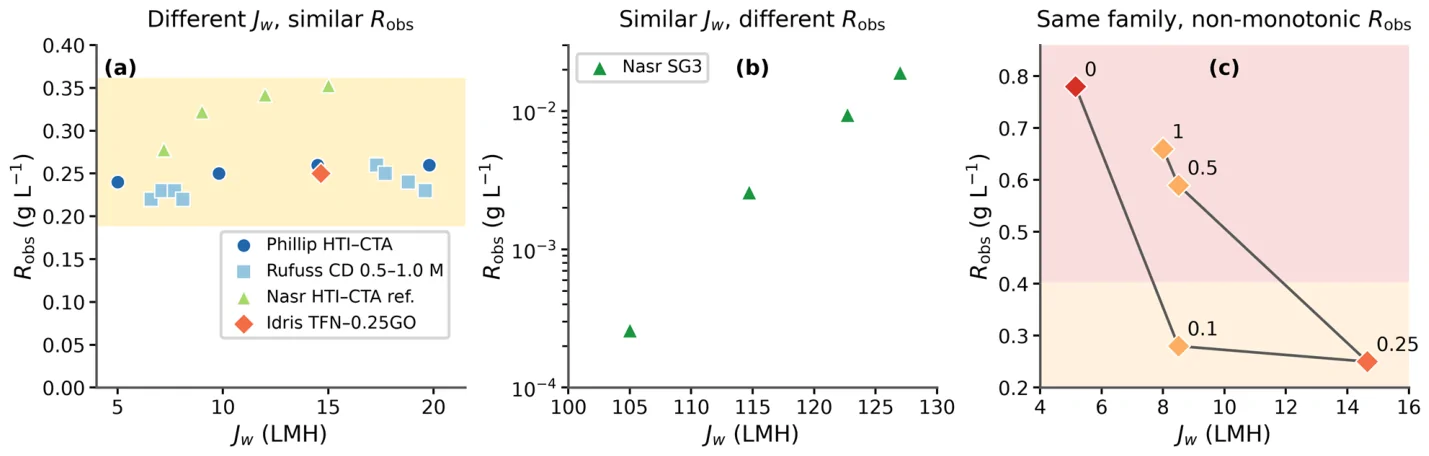
Observable equivalence and transport-state divergence in FO transport space. (**a**) Membrane systems with comparable measured *R_obs_* values but different water fluxes. The yellow shaded band denotes the declared transitional range (**b**) The Nasr SG3 series combines high water flux with leakage ratios spanning almost two orders of magnitude. (**c**) Non-monotonic trajectory of the TFN-GO family as GO loading changes. The pale-ochre and pale-red shading denote the transitional and leakage-dominated ranges, respectively. All plotted coordinates are measured or derived values from Table S2.

The workflow described in Section 2.6 was applied to four published FO datasets [4,14,15,16]. The reported *J_w_* and *J_s_* values were converted to common units, and *R_J_* was calculated from Equation (1). The resulting observations define the distributions shown in Figure 1 and Figure 2 and provide the input for the regime-weight, concentration-polarization, compatibility, and rank-aware analyses in Section 3.3, Section 3.4, Section 3.5 and Section 3.6. The dataset includes commercial cellulose triacetate membranes, aquaporin-based hollow-fiber membranes [14], TFN-GO membranes, and PES/GO mixed-matrix membranes [15,16]. Aquaporin-based biomimetic membrane architectures are reviewed more generally in [24]. Across the 26 observations listed in Table 3 and Table S2, *J_w_* ranges from 5.0 to 127.0 LMH and *R_J_* from 2.6 × 10^−4^ to 0.80 g L^−1^.

Phillip et al. reported HTI-CTA membrane performance with NaCl draw solutions at concentrations ranging from 1 to 4 M. Water flux increased from approximately 5 to 20 LMH across this range, while *R_obs_* remained nearly stable at 0.24–0.26 g L^−1^, confining this dataset to a narrow transitional interval of moderate leakage normalized to water productivity.

The Aquaporin HFFO.6 hollow-fiber data of Rufuss et al. [14] span a wider operational range, incorporating variations in draw concentration, temperature, and flow rate. *J_w_* extends from approximately 6.6 to 33 LMH and *R_obs_* from approximately 0.22 to 0.80 g L^−1^, tracing a trajectory from the transitional region toward leakage-dominated behavior.

The PES/GO SG3 membrane reported by Nasr et al. occupies the high-flux, low-leakage region, with *J_w_* = 105.0–127.0 LMH and *R_J_* = 2.6 × 10^−4^–1.9 × 10^−2^ g L^−1^ [15]. The HTI-CTA reference from the same study remains in the transitional region, with *J_w_* = 7.2–15.0 LMH and *R_J_* = 0.28 − 0.35 g L^−1^.

Idris et al. examined TFN membranes containing 0–1.0 wt% GO [16]. The unmodified TFN-0GO membrane had *J_w_* = 5.15 LMH and *R_J_* = 0.78 g L^−1^. Addition of 0.25 wt% GO increased *J_w_* to 14.65 LMH and reduced *R_J_* to 0.25 g L^−1^. Further increases in GO loading shifted the response toward higher leakage ratios: *R_J_* = 0.59 g L^−1^ at 0.5 wt% GO and 0.66 g L^−1^ at 1.0 wt% GO.

The observations cluster preferentially within the three defined *R_J_* ranges, but the boundaries between regimes are not sharp physical thresholds. The continuous weights of Equations (4)–(6) assign gradual compatibility values around the adopted *R_S_* and *R_L_* values without assigning a unique transport mechanism to each point, which is particularly relevant in the transitional region where comparable (*J_w_*, *R_J_*) coordinates remain compatible with different combinations of membrane and operating-condition effects. The observable overlap visible in Figure 1 reflects both transport heterogeneity and concentration-polarization amplification and also the rank-two structure of the observable representation: the same region of the (*J_w_*, *R_J_*) plane can remain compatible with multiple transport-support organizations because *J_w_*, *J_s_*, and *R_J_* do not constrain all latent transport directions independently. Dashed curves in Figure 1 show illustrative constant- *R*_0_ trajectories from Equation (3) evaluated at *k*_eff_ = 40 LMH; they are not fitted contours or uncertainty bounds.

Figure 1 therefore provides an empirical identifiability map rather than a membrane-classification diagram. Its scientific message is that the observable position of a membrane is not uniquely associated with a specific underlying transport mechanism. Separation in the (*J_w_*, *R_J_*) plane demonstrates that two conditions differ in measured transport performance; however, proximity or overlap in the same plane demonstrates that distinct membrane architectures, compositions, or operating conditions may become experimentally indistinguishable when they are observed only through *J_w_* and *R_J_*. The reader can therefore use Figure 1 to identify where the available measurements discriminate performance states and, equally importantly, where they fail to discriminate their physical origin. In those overlapping regions, the data alone cannot establish whether the observed response is governed primarily by selective-layer permeability, support resistance, concentration polarization, or defect-mediated leakage. Figure 1 thus provides the direct experimental evidence for the non-unique observable-to-mechanism mapping that is subsequently quantified by the admissible-region and rank analyses.

### 3.2. Observable Equivalence and Non-Identifiability in FO Transport Space

Figure 2 compares observations that illustrate three forms of non-unique interpretation: similar *R_J_* values obtained with different membrane systems or water fluxes, high-flux responses associated with markedly different leakage penalties, and a non-monotonic trajectory within the Idris TFN-GO series.

Figure 2 compares membrane systems selected because they occupy nearby *R_J_* values in the (*J_w_*, *R_J_*) observable plane, although they differ in membrane architecture and operating conditions.

The Phillip HTI-CTA observations remain near *R_J_* ≈ 0.24–0.26 g L^−1^ over *J_w_* ≈ 5–20 LMH, whereas the Idris TFN-0.25GO membrane reaches *R_J_* = 0.25 g L^−1^ at *J_w_* = 14.65 LMH. Thus, proximity in measured observable coordinates is compatible with substantial diversity in membrane architecture, support structure, and operating conditions.

Figure 2b considers membrane systems with high water productivity but different leakage-normalized responses. The Nasr SG3 membrane combines *J_w_* = 105.0–127.0 LMH with *R_J_* = 2.6 × 10^−4^–1.9 × 10^−2^ g L^−1^, whereas the Rufuss HFFO.6 observation at the highest draw concentration reaches *J_w_* = 33.0 LMH and *R_J_* = 0.80 g L^−1^. High water flux can therefore be associated with very different reverse-solute penalties, depending on membrane properties and operating conditions.

Figure 2c shows that the trajectory within a single membrane family can also be non-monotonic. In the Idris TFN-GO series, addition of 0.25 wt% GO decreases *R_J_* relative to TFN-0GO, whereas further increases to 0.5 and 1.0 wt% shift the response back toward higher leakage ratios. A material modification therefore does not correspond to a unique direction in the (*J_w_*, *R_J_*) plane.

Together, the three comparisons show that structurally different membranes can occupy neighboring observable regions and that a single material series can traverse the same space non-monotonically. These observations motivate the continuous regime weights of Section 3.3 and the compatibility and rank analyses developed in Section 3.5 and Section 3.6.

### 3.3. Soft Regime Occupancy and Continuous Transitions

The soft regime weights defined in Section 2.4 were evaluated at the *R_J_* values reported in Section 3.1. No additional fitting was performed. The functions *P_S_*, *P_T_*, and *P_L_* describe compatibility with selective, transitional, and leakage-dominated transport, respectively. *P_S_* dominates below *R_S_*, *P_L_* increases above *R_L_*, and the residual weight *P_T_* is largest over the intermediate range. The three normalized weights therefore replace categorical assignment with a continuous representation of regime compatibility.

**Figure 3:**
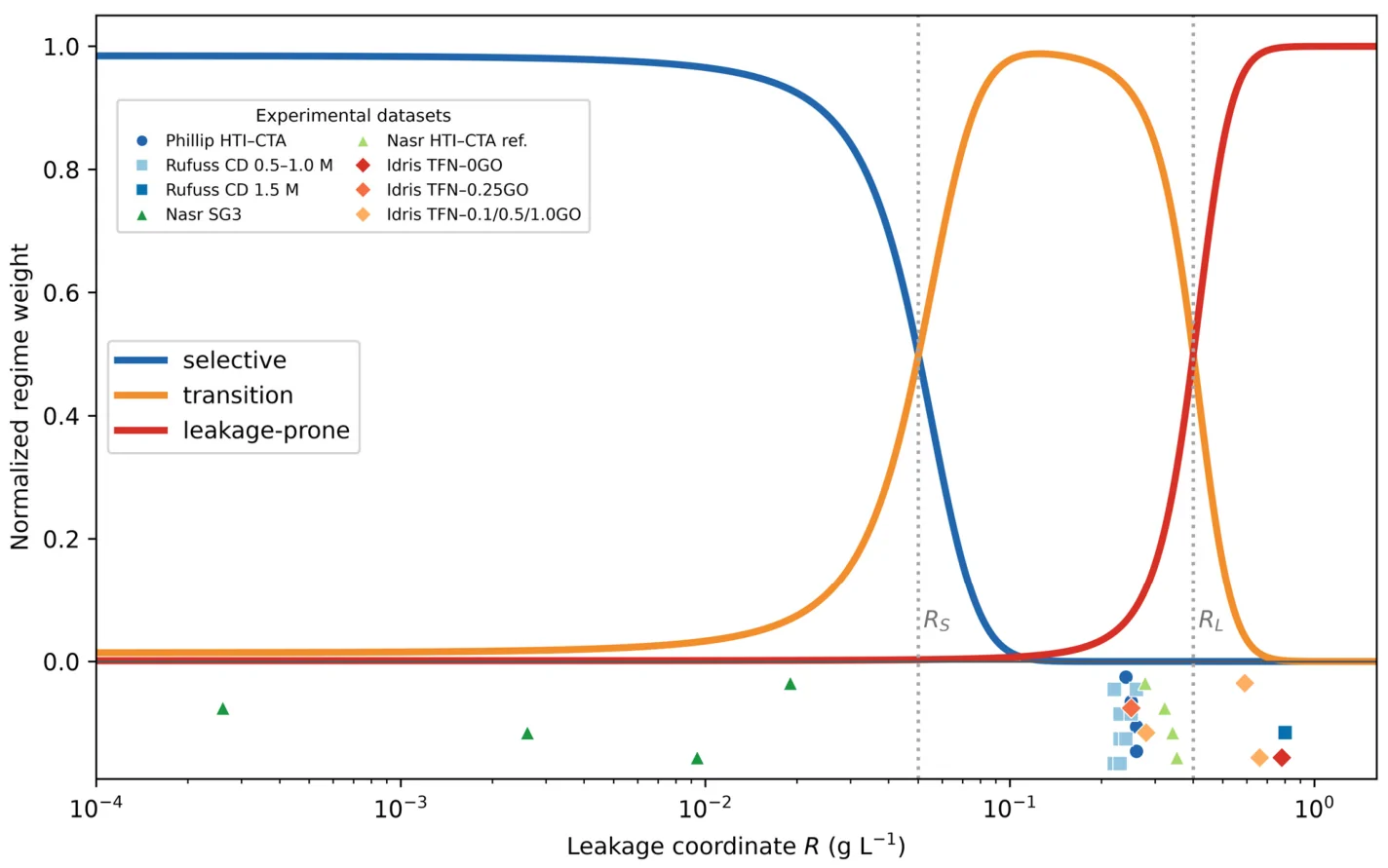
Continuous admissibility weights and experimental positions in the reverse-solute-ratio coordinate. The selective, transitional, and leakage-dominated weights *P_S_*, *P_T_*, and *P_L_* were calculated from Equations (4)–(6) and are shown as functions of *R_CP_* on a logarithmic scale. Experimental observations are positioned along the lower axis using their measured ratios *R_obs_* = *J_s_*/*J_w_*. Marker symbols identify the eight experimental datasets, as shown in the legend of Figure 1. The grey dashed vertical lines mark the regime reference coordinates (*R_L_* = 0.05 and *R_L_* = 0.40 g L^−1^).

Figure 3 places the experimental *R_obs_* values on the same logarithmic axis as the continuous regime weights. Three of the four Nasr SG3 observations lie below 10^−2^ g L^−1^, and the fourth is 1.9 × 10^−2^ g L^−1^; all four remain within the selective region defined by *R_S_*. The lowest SG3 value, 2.6 × 10^−4^ g L^−1^, is nearly three orders of magnitude below the transitional cluster near 0.22–0.35 g L^−1^, whereas the highest SG3 value is approximately one order of magnitude lower.

The weights are continuous compatibility functions, not probabilities of uniquely identified mechanisms. For legibility, experimental observations are shown at vertically offset display levels within the same panel; their horizontal positions are the measured *R_obs_* values and are not jittered.

The Phillip HTI-CTA, Rufuss low-CD, Nasr HTI-CTA reference, and Idris TFN-0.25GO observations occupy the transitional region. The Rufuss measurement at 1.5 M draw concentration lies in the leakage-dominated region. Its displacement relative to the lower-concentration HFFO.6 observations is compatible with an operating-condition-dependent increase in apparent leakage, although the available observables do not identify concentration polarization as its unique cause [2,5]. The vertical lines in Figure 3 indicate the adopted regime thresholds; the logistic functions describe continuous transitions around these values.

### 3.4. Concentration-Polarization Amplification of Observable Ambiguity

**Figure 4:**
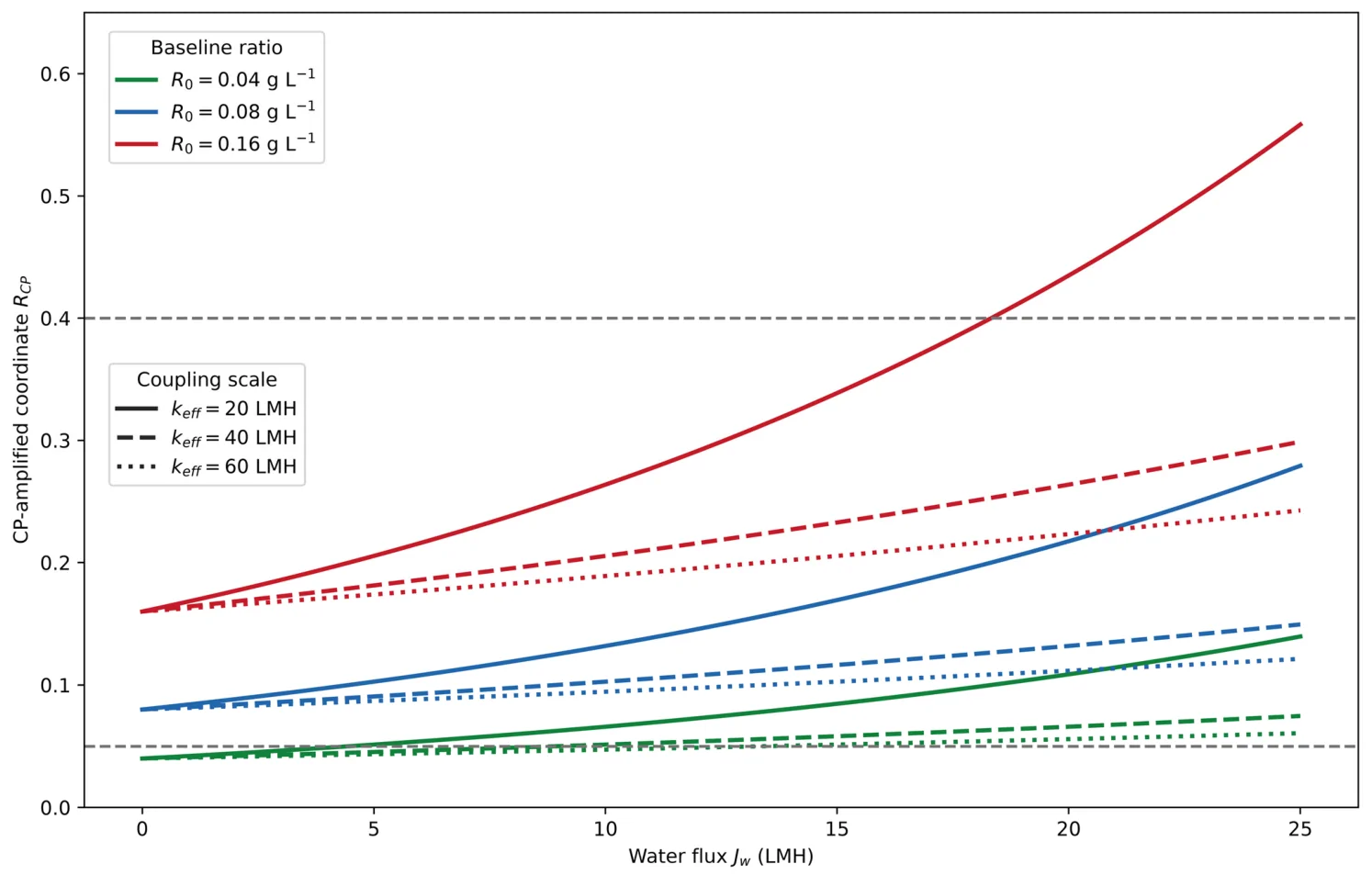
Concentration-polarization amplification of the observable leakage ratio. Curves show *R_CP_* = *R*_0_ exp(*J_w_*/*k_eff_*) for *R*_0_ = 0.04, 0.08, and 0.16 g L^−1^ and *k_eff_* = 20, 40, and 60 LMH. Horizontal lines denote the declared regime coordinates *R_S_* = 0.05 and *R_L_* = 0.40 g L^−1^. Colors distinguish the three baseline ratios and line styles distinguish the three coupling scales. These are illustrative reduced-model trajectories, not fitted membrane reconstructions. The horizontal grey dashed lines denote the declared regime coordinates at 0.05 and 0.40 g L^−1^.

Figure 4 applies the concentration-polarization amplification relation of Section 2.3 to representative baseline ratios *R*_0_ while varying *k*_eff_ [2,5]. The calculated curves show how *R*_CP_ increases with *J_w_* and how the increase becomes stronger as *k*_eff_ decreases. Under this reduced model, a baseline response can move from the selective range toward the transitional or leakage-dominated range without requiring a change in *R*_0_. *k*_eff_ functions here as a phenomenological amplification scale. It summarizes the sensitivity of the apparent leakage ratio to water flux under polarization-related coupling but does not separate external polarization, internal polarization, support resistance, and defect-mediated leakage into independently estimated contributions. Figure 4 therefore examines how uncertainty in this effective coupling changes the predicted observable response; the curves are illustrative model trajectories rather than fitted reconstructions of individual membranes.

The persistence of admissible regions in the structural-support landscape indicates that inverse ambiguity is not generated solely by membrane heterogeneity or concentration-polarization amplification. A complementary question is whether the commonly reported observables contain enough independent information to constrain all relevant transport-support directions. This question is addressed directly in the rank-aware analysis of Section 3.6.

### 3.5. Admissible Structural-Support Landscape

The measured (*J_w_*, *R_J_*) response is next mapped onto the reduced (*A*, *f_d_*) plane defined in Section 2.5. *A* represents effective productive permeability, whereas *f_d_* represents defect-mediated leakage. Both are reduced model descriptors rather than direct measurements of unique membrane properties. For each parameter pair, the forward model predicts *J_w_* and *R_J_*. Equation (7) then compares these predictions with the observed response using the uncertainty widths $\sigma_{J_{w}}$ and $\sigma_{R_{J}}$.

**Figure 5:**
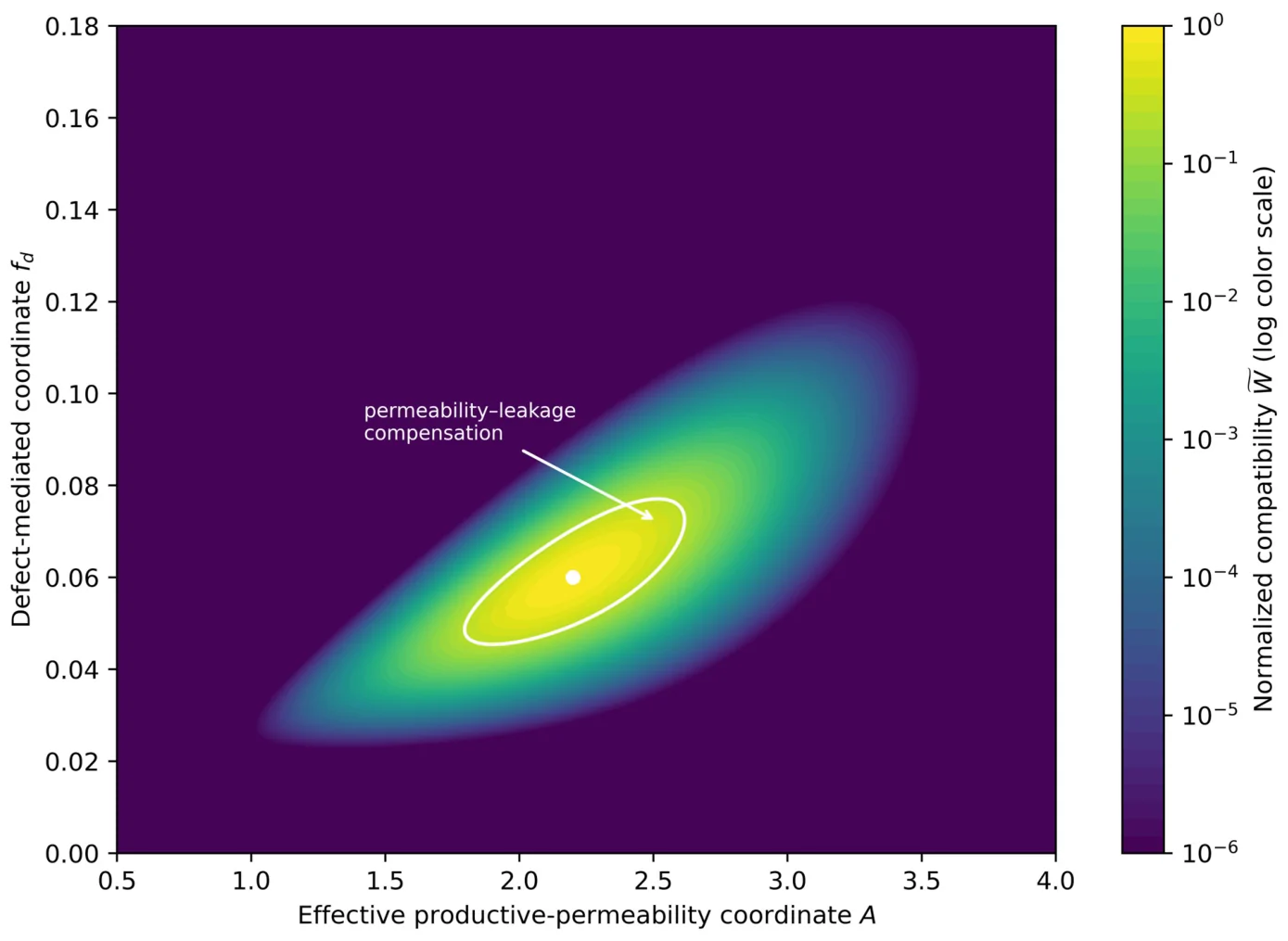
Compatibility landscape in the reduced (*A*, *f_d_*) structural-support plane. The normalized field $\tilde{W}$ is evaluated at the median observable point and displayed on a logarithmic color scale. The white contour denotes the admissibility threshold $\tilde{W}$ = 0.22, and the marker denotes the reference coordinate. The finite contour shows that the flux observables constrain the explored parameter space, while its elongation identifies a weakly resolved permeability–leakage compensation direction. The positive slope reflects inverse-problem compensation and does not imply a causal relation between permeability and defect formation. Complete settings and gridded values are provided in the Supplementary Materials.

Figure 5 shows the normalized compatibility landscape at the median observable point of the harmonized dataset. Calibration of the reduced forward map places the reference maximum at *A* = 2.2 and *f_d_* = 0.06. The white contour marks the reference admissibility threshold *W_thr_* = 0.22; all numerical settings and the complete gridded field are supplied with the Supplementary Materials.

The admissible region is finite but elongated, with a positive association. This geometry has a direct physical and inferential meaning. The finite extent shows that the measured flux observables do constrain the reduced transport-support space: large portions of the explored (*P_T_*, *D_f_*) domain are incompatible with the reference response. However, the elongated direction shows that this constraint is strongly anisotropic and does not permit unique identification. Along this direction, an increase in effective productive permeability can be compensated by an increase in defect-mediated leakage while the predicted *J_w_* and *R_J_* remain within the adopted uncertainty envelope. The positive slope is therefore not interpreted as evidence of a physical causal relation between permeability and defect formation; it identifies a compensation direction generated by the inverse mapping from the available observables to the reduced parameter space. Such elongated admissible regions are characteristic of parameter combinations that are only weakly distinguishable by the available measurements in inverse and identifiability analyses [12,13,25]. The scientific information gained from Figure 5 is therefore twofold: the conventional flux observables exclude a substantial fraction of possible transport-support states, but they leave a specific permeability–leakage direction unresolved. The location of the compatibility maximum is consequently less informative than the geometry and extent of the admissible region. Across the 26 observations, the median admissible-volume fraction is 1.19 × 10^−2^. Because this value depends on the declared reduced model, uncertainty widths, threshold, grid, and parameter bounds, it is used as a comparative measure of remaining ambiguity and not as an intrinsic membrane property. Independent measurements sensitive to permeability, support/polarization behavior, or defect-mediated transport are therefore required to collapse the unresolved direction and distinguish among states that remain compatible with the same flux response.

The information gain relative to a conventional flux plot therefore lies in identifying the unresolved compensation direction, not in reconstructing a unique membrane state. In the present reduced representation, this direction corresponds to the trade-off between productive permeability and leakage. Independent permeability measurements, defect-sensitive structural characterization, and orientation-dependent experiments provide examples of the additional physical information needed to constrain such unresolved directions [17,18,19].

### 3.6. Observable-Rank Limitation and Admissible Compatibility Structure

**Figure 6:**
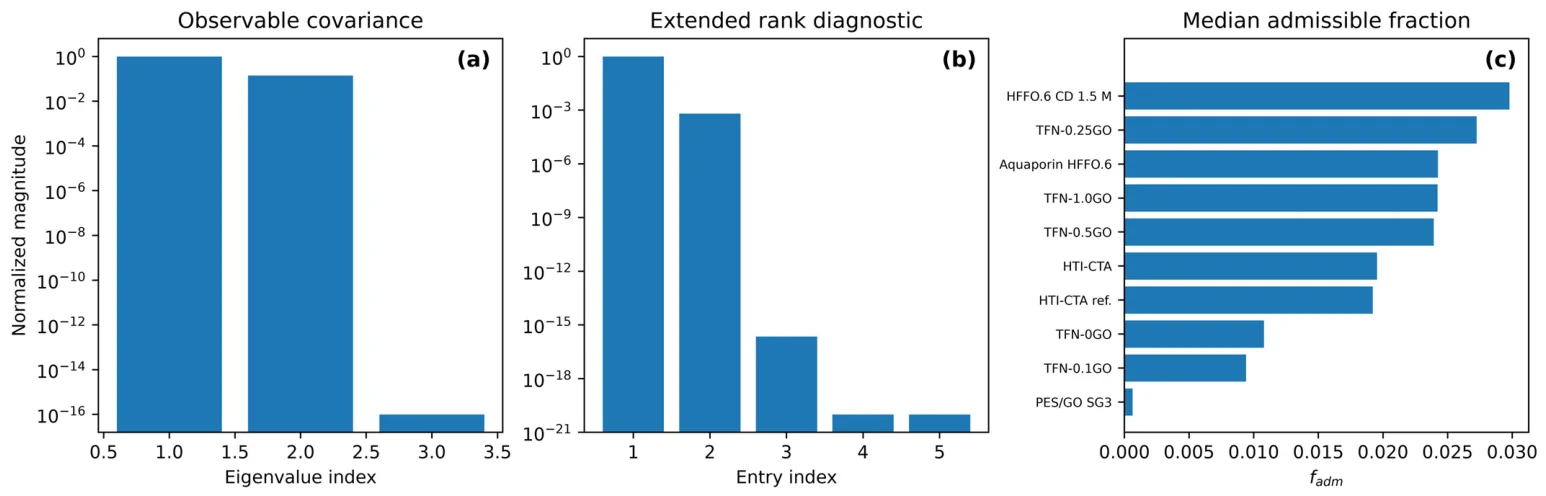
Observable rank and residual compatibility. (**a**) Normalized covariance eigenvalues of (log *J_w_*, log *J_s_*, log *R_J_*), with *R_J_* recomputed from *J_w_* and *J_s_* for the rank audit. (**b**) Extended five-entry rank diagnostic: two uncertainty-scaled local Fisher eigenvalues, one algebraic-null scale, and two explicitly constructed structural-null entries. (**c**) Median observation-level admissible-volume fraction for each membrane system at *W_thr_* = 0.22. Numerical values and construction details are provided in Tables S5–S7.

Figure 6 presents the rank-aware consistency check described in Section 2.7 through three coordinated panels, each addressing a distinct aspect of the information available in the experimental dataset.

Figure 6a shows the normalized covariance eigenvalues of (log *J_w_*, log *J_s_*, log *R_J_*) for the 26 observations. The first two values are 1.000 and 1.39 × 10^−1^; the magnitude of the third is 5.64 × 10^−17^ and is numerically zero. The third value follows from log *R_J_* = log *J_s_* − log *J_w_* when *R_J_* is recomputed from the same flux columns, confirming an effective observable rank of two.

Figure 6b reports an explicitly constructed five-entry extended rank-diagnostic spectrum. The first two entries, 1.000 and 6.46 × 10^−4^, are the normalized eigenvalues of the uncertainty-scaled local Fisher matrix. The third entry is the machine-precision algebraic-null scale of the observable representation; the final two entries are structural zero-padding used only to display the rank-two mapping in the five-entry diagnostic space. They are not additional fitted eigenvalues or independently estimable parameters.

The five plotted entries describe a rank-two mapping embedded in an extended diagnostic representation; they do not imply five independently estimable parameters. The separation between the second entry (6.46 × 10^−4^) and the algebraic-null scale (2.22 × 10^−16^) is displayed directly without imposing an additional numerical-rank threshold.

Figure 6c reports the median observation-level admissible-volume fraction for each membrane system at *W_thr_* = 0.22. Values span 6.28 × 10^−4^ for PES/GO SG3 to 2.98 × 10^−2^ for HFFO.6 CD 1.5 M. This variation reflects the position of each response relative to the declared model domain and resolution settings; it should not be interpreted as a direct measure of membrane heterogeneity.

For example, the system-level medians are 1.95 × 10^−2^ for Phillip HTI-CTA and 6.28 × 10^−4^ for Nasr PES/GO SG3. The median across all 26 observation-level fractions is 1.19 × 10^−2^. The complete values and aggregation rule are reported in Table S5 and the accompanying CSV file.

Taken together, the three panels of Figure 6 show that observable ambiguity in FO characterization has two separable origins. The algebraic rank deficiency documented in Figure 6a is universal and cannot be reduced by adding more single-condition flux measurements; it disappears only when structurally independent observables are introduced as quantities not derivable from *J_w_* and *J_s_* alone. The forward-model insensitivity documented in Figure 6b reflects the reduced transport-support parameter space and the limited projection that two independent observables can provide onto a higher-dimensional transport-support description. The per-dataset admissible fractions in Figure 6c show that this joint effect has different practical magnitudes across the experimental dataset, providing a system-specific criterion for assessing where additional measurements would most effectively reduce residual ambiguity.

### 3.7. Experimental Cases with Complementary Physical Information

In the AL-FS series of Kallem et al. [17], modification from the pristine TFC-PES membrane to TFC-PES/PDA@TiO2 increased *J_w_* from 12.5 to 34.3 LMH and *J_s_* from 6.1 to 9.2 g m^−2^ h^−1^, while *R_J_* decreased from 0.488 to 0.268 g L^−1^. Independent characterization of the same membrane series reported an increase in water permeability from 1.62 to 3.19 and a smaller change in salt permeability from 1.11 to 1.23, with the corresponding permeability ratio decreasing from 0.68 to 0.38.

Bai and Kruczek [19] examined the same commercial osmotic membrane in AL-FS and AL-DS configurations. Their dynamic measurements reported a salt-transport time lag of approximately 4.3–4.6 min in AL-FS, whereas in AL-DS it increased from about 7 to 20 min with draw concentration; the water response was also orientation dependent. He et al. [18] combined FO transport measurements with morphological and structural characterization of polyamide layers produced under different interfacial-polymerization conditions, with the lower reverse-solute-transport membrane showing a denser and more continuous selective-layer morphology with reduced defect formation.

## 4. Discussion

### 4.1. Coherence Loss and Observable Insufficiency

FO membrane interpretation is constrained by two coupled factors: the physical degeneracy of transport mechanisms and the rank-limited information content of the available observables. Accordingly, the analysis first establishes the information content of the measured observables, then maps the transport states compatible with those observations, and finally examines whether complementary independent measurements can constrain the remaining mechanistic ambiguity. This distinction is essential because conventional FO flux data are commonly used both to compare membrane performance and to infer the physical origin of that performance. The first task can be addressed directly from the measured fluxes, whereas the second may remain non-identifiable from the same dataset. Because *R_J_* = *J_s_*/*J_w_*, reporting *R_J_* together with *J_w_* and *J_s_* does not introduce a third independent observable direction; improved precision or denser sampling of the same quantities can therefore reduce numerical uncertainty without eliminating mechanistic degeneracy. Therefore, changes associated with selective-layer permeability, support resistance and concentration polarization, or defect-mediated leakage may be attributed to a specific physical cause even when alternative mechanisms remain equally compatible with the observed response. An explicit identifiability analysis is therefore required to determine which mechanistic conclusions are supported by the available flux data and which require additional independent transport or structural measurements.

At the physical level, water permeation, reverse-solute transport, concentration polarization, support resistance, and defect-mediated pathways can compensate one another in the measured response [1,2,3,4,5,6,26,27]. At the informational level, *J_w_*, *J_s_*, and *R_J_* provide only a low-dimensional projection of this coupled transport reality. The framework therefore treats *R_J_* as a derived observable coordinate that summarizes the balance between reverse-solute flux and water flux, carrying informational content distinct from that of an independently measured structural parameter.

The direct consequence for data interpretation is that a high *R_J_* value restricts the admissible transport-support configurations toward leakage-dominated states, while a low *R_J_* value is consistent with a range of selective-layer conditions whose structural origin requires additional independent measurements to resolve. Through the admissible set 𝒜(D) defined in Equation (2), each measurement defines a compatibility class of plausible transport-support mechanisms.

Figure 1 and Figure 2 provide the experimental basis for this interpretation. The selected systems span *R_J_* values from 2.6 × 10^−4^ g L^−1^ for the lowest Nasr SG3 observation to 0.78 g L^−1^ for Idris TFN-0GO. Phillip HTI-CTA remains near *R_J_* = 0.24–0.26 g L^−1^ over *J_w_* = 5.0–19.8 LMH, whereas Idris TFN-0.25GO reaches *R_J_* = 0.25 g L^−1^ at *J_w_* = 14.65 LMH. These observations occupy neighboring regions of the (*J_w_*, *R_J_*) plane despite their different compositions and architectures. Their corresponding admissible sets may overlap under the adopted uncertainty model, but they are not the same observation *D*. Figure 3 further shows that compatibility with the defined transport regimes changes continuously around the adopted thresholds.

The present analysis operates at three levels that are structurally connected rather than merely sequential. Classical FO transport equations supply the mechanistic relations between water flux, solute flux, membrane permeability, support effects, and concentration polarization. Observable-space organization translates these relations into a physically grounded representation of sparse experimental data. The rank-aware admissibility layer then quantifies which transport-support directions the available observables actually constrain and which they leave open.

### 4.2. Admissible Inference Under Observable-Rank Limitation

The coherence-loss analysis by Viviani et al. [20] provides the physical justification for using *R_J_* as a simplified transport descriptor. The present approach shifts the analytical target toward identifying, through Equation (2), the transport-support states that remain consistent with the measured observables rather than merely classifying transport deterioration into regimes. Diagnostic classification and admissible inference are therefore complementary: the former organizes observable behavior, the latter characterizes the ambiguity that a limited observable set leaves unresolved.

The concentration-polarization trajectories in Figure 4 quantify the sensitivity of *R*_CP_ to the effective coupling scale in Equation (3). For example, at *J_w_* = 20 LMH and *R*_0_ = 0.08 g L^−1^, the calculated *R*_CP_ values are 0.22, 0.13 and 0.11 g L^−1^ for *k*_eff_ = 20, 40, and 60 LMH, respectively. The coupling scale therefore changes the predicted position within the transitional region, although the magnitude of the displacement depends on both *R*_0_ and *J_w_*. This sensitivity broadens the range of effective transport conditions compatible with an observed response without, by itself, demonstrating a change in selective-layer properties.

### 4.3. Reduced Physics-Informed Compatibility Assessment

Figure 5 provides the central inferential result of the framework by identifying the (*A*, *f_d_*) combinations that remain compatible with the reference observable response under the adopted uncertainty model. The finite and elongated high-weight region shows that identifiability should be evaluated explicitly when more complex inverse methods are applied. Bayesian inversion and physics-informed machine-learning approaches can represent this uncertainty when their likelihoods, priors, parameterizations, and observable structures are formulated accordingly. Without those provisions, numerical concentration of the inferred output may overstate the independent information contained in the measurements [28,29,30,31]. The median admissible-volume fraction is not highly sensitive to this choice: across *W_thr_* = 0.10–0.40, the observation-level median varies only from 1.68 × 10^−2^ to 6.66 × 10^−3^ (Table S1), so the compensation pattern in Figure 5 is not an artifact of the specific threshold adopted. 

For the Nasr SG3 series, *R_J_* ranges from 2.6 × 10^−4^ to 1.9 × 10^−2^ g L^−1^, and the corresponding *P_S_* values place the observations predominantly within the selective region. For Idris TFN-0GO, *R_J_* = 0.78 g L^−1^ and *P_L_* approaches 1, placing the observation within the leakage-dominated region. In the reduced (*A*, *f_d_*) representation, these contrasting observable responses shift the corresponding high-weight regions toward different *f_d_* ranges. In both cases, however, the finite width of the region indicates that the available *J_w_* and *R_J_* constraints do not identify a unique parameter pair.

The framework occupies an intermediate level between empirical performance mapping and full parameter inversion. Relative to a conventional *J_w_*–*R_J_* plot, it introduces explicit transport coupling, concentration-polarization amplification, uncertainty widths, and a reduced compatibility map. Relative to full inversion, it limits interpretation to the parameter combinations supported by the available observable basis. This level of analysis is suited to comparative FO datasets in which *J_w_* and *J_s_*, and consequently *R_J_*, are the principal reported transport measurements.

### 4.4. Measurement Design Implications of Rank-Aware Admissibility

Figure 6 provides a quantitative consistency check for the admissible-overlap analysis. Panel a confirms the exact rank-two observable structure; panel b distinguishes the two local sensitivity directions from algebraic and constructed null entries; and panel c summarizes the conditional extent of the admissible domain. Together these diagnostics show why improved precision within the existing flux coordinates can narrow compatibility regions without creating an additional independent observable direction.

Additional measurements are most informative when they constrain directions that are not independently supported by the existing observables [32,33,34]. Repeated *J_w_* and *J_s_* measurements under the same conditions can reduce uncertainty in those coordinates but do not increase their algebraic rank. Five complementary measurement classes could reduce the residual admissible region.

Independent salt-permeability measurements *B*, obtained using an appropriate pressure-driven or diffusion-based protocol, would constrain selective-layer solute transport separately from the combined FO response. Independent determination of the structural parameter *S*, through measurements of support thickness, porosity, and tortuosity or through a validated transport experiment, would constrain support-related internal concentration polarization. Tracer experiments using solutes of known diffusivity could distinguish defect-mediated leakage from the bulk water-flux response. Comparisons between active-layer-facing-feed (AL-FS) and active-layer-facing-draw (AL-DS) orientations under matched draw and feed conditions would probe the orientation dependence of internal concentration polarization. Finally, measurements across several draw concentrations would provide a transport-response trajectory rather than a single operating point and could help separate baseline leakage from flux-dependent amplification.

These measurements address different unresolved directions in the present analysis. Figure 5 shows that the flux observables can restrict the admissible region while still leaving a permeability–leakage compensation direction unresolved. Figure 6b expresses the same limitation locally through the strongly unequal Fisher eigenvalues, whereas Figure 6c indicates how much of the declared parameter domain remains compatible with each observation. The framework therefore identifies both where ambiguity remains and the physical direction along which it occurs. It does not, however, determine which additional experiment should be performed: that choice depends on the mechanism associated with the unresolved direction.

The three experimental cases show how this can occur in practice. Kallem et al. [17] provide independent permeability measurements that distinguish whether a favorable change in FO performance is associated primarily with water transport or with solute transport. Bai and Kruczek [19] show that changing membrane orientation modifies the transport response even when the membrane itself is unchanged, thereby identifying support and polarization effects that cannot be separated from flux measurements alone. He et al. [18] provide independent structural evidence on the selective layer, allowing suppressed reverse-solute transport to be associated with reduced defect formation rather than inferred from the flux ratio alone. In each case, the complementary measurement adds physical information absent from the conventional flux observables and therefore reduces the mechanistic ambiguity identified by the admissible-region analysis.

### 4.5. Implications for Membrane Design and AI

For membrane design, the framework is most useful as a screening tool rather than as a unique inverse-design method. A target region in the (*J_w_*, *R_J_*) plane can be specified from the desired productivity and leakage penalty, and the associated admissible region then shows which combinations of productive transport and leakage remain compatible with that target. A broad or elongated region signals that design decisions should be supported by an additional measurement directed at the unresolved mechanism—for example, support/ICP characterization when polarization is suspected, or salt-permeability and selective-layer measurements when leakage remains ambiguous.

AI-based methods may support this framework by accelerating forward calculations, screening large parameter spaces, or integrating flux data with additional membrane and operating descriptors [33,34,35]. Ibrar et al. [35], for example, used supervised learning to predict reverse-solute flux from membrane and operating variables. In the present context, the main benefit of such approaches is not to replace independent physical characterization, but to exploit it more efficiently when it is available.

### 4.6. Limitations

The framework has several limitations. The primary harmonized dataset remains limited to 26 observations from four published studies, and several reverse-solute-flux values were digitized from reported figures rather than tabulated directly by the source authors (marked with † in Table S2), which adds a secondary source of numerical uncertainty beyond that of the original measurements. The compatibility widths and the regime thresholds *R_S_* and *R_L_* are declared analysis settings rather than quantities fitted to or independently derived from the data; the sensitivity checks in Section 2.7 and Supplementary Materials Section S2.4 show that the qualitative conclusions are robust to reasonable variation in these settings, but the reported admissible-volume fractions remain conditional on them. Finally, the reduced forward model of Section 2.5 is a two-parameter simplification intended to demonstrate the admissible-inference approach; it is not a mechanistic membrane transport model and should not be used to infer specific structural parameters beyond the compatibility regions it defines.

## 5. Conclusions

This study establishes an observable-space framework for determining how far conventional FO measurements can support mechanistic interpretation of membrane transport. The need for such an analysis arises because the same flux data that reliably describe membrane performance may remain insufficient to identify its physical origin: different combinations of selective-layer permeability, support resistance, concentration polarization, and defect-mediated leakage can produce similar observable responses. The (*J_w_*, *R_J_*) representation, concentration-polarization amplification model, continuous regime weights, rank analysis, and compatibility mapping provide a unified means of identifying and quantifying this residual non-uniqueness.

The observable triplet (*J_w_*, *J_s_*, *R_J_*) contains only two independent scalar quantities because *R_J_* = *J_s_*/*J_w_*. Consequently, the remaining ambiguity reflects both compensating transport mechanisms and the limited independent information contained in the measurements. Increasing the precision or density of the same flux observations can reduce numerical uncertainty but cannot by itself introduce an additional mechanistic constraint. Within the declared reduced model, uncertainty assumptions, admissibility threshold, and parameter domain, the admissible-volume fraction provides a quantitative measure of the transport states that remain compatible with the observed response.

More specific mechanistic inference therefore requires measurements that constrain physical directions not contained in the flux pair itself, including selective-layer permeability, support and concentration-polarization characteristics, defect-sensitive structural information, or transport responses acquired under independently varied operating conditions. The practical value of the framework is thus twofold: it determines how strongly existing FO measurements constrain a reduced transport description and indicates when an additional independent measurement is required before a more specific physical interpretation can be justified. The three experimental case studies illustrate this principle by showing how complementary permeability, orientation-dependent transport, and selective-layer characterization can resolve ambiguities that remain inaccessible to flux-only analysis.

## Figures and Tables

**Table 2:** Principal observable, effective, and latent quantities used in the admissible inference framework.

Symbol	Definition	Status in the Framework	Interpretation
*J_w_*	Water flux	Measured or literature-extracted	Productivity observable
*J_s_*	Reverse-solute flux	Measured or literature-extracted	Leakage observable
*R_J_*	*J_s_*/*J_w_*	Derived observable	Coherence-sensitive transport ratio
*R_CP_*	CP-amplified ratio	Effective observable	Apparent leakage under CP amplification
*k*_eff_	Effective CP coupling scale	Effective parameter	Strength of CP-mediated amplification
*P_S_*	Stable-state occupancy	Soft admissibility weight	Compatibility with selective transport
*P_T_*	Transitional occupancy	Soft admissibility weight	Compatibility with intermediate transport
*P_L_*	Leakage-state occupancy	Soft admissibility weight	Compatibility with leakage-dominated transport
*A*	Effective permeability descriptor	Reduced coordinate	Productive transport support
*f_d_*	Effective defect descriptor	Reduced coordinate	Defect-mediated leakage support

Note: the standard FO observables and the specific reverse-solute ratio follow established FO usage [1,4,20], whereas the concentration-polarization basis follows [2,5]. The soft admissibility weights, reduced coordinates, and compatibility construction are introduced in the present work.

**Table 3:** Harmonized experimental coordinates used in this study.

Source	Membrane	Draw Solution	*J_w_* (LMH)	*R_J_* (g L^−1^)
Phillip et al. [4].	HTI-CTA	1–4 M NaCl	5–20	0.24–0.26
Rufuss et al. [14].	Aquaporin HFFO.6	0.5–1.0 M NaCl	6.6–19.6	0.22–0.26
Rufuss et al. [14].	Aquaporin HFFO.6	1.5 M NaCl	22.3–33	0.42–0.80
Nasr et al. [15].	PES/GO SG3	0.65–2 M NaCl	105–127	2.6 × 10^−4^– ∼1.9 × 10^−2^
Nasr et al. [15].	HTI-CTA (ref.)	0.65–2 M NaCl	7.2–15	0.28–0.35
Idris et al. [16].	TFN-0GO	1 M NaCl	5.15	0.78
Idris et al. [16].	TFN-0.1GO	1 M NaCl	~8.5	~0.28
Idris et al. [16].	TFN-0.25GO	1 M NaCl	14.65	0.25
Idris et al. [16].	TFN-0.5GO	1 M NaCl	~8.5	~0.59
Idris et al. [16].	TFN-1.0GO	1 M NaCl	~8.0	~0.66

*J_w_* is reported in LMH, and *R_J_* = *J_s_*/*J_w_* is reported in g L^−1^. Values marked with ∼ were approximated from published figures and are used only for illustrative admissibility mapping. Proportional uncertainty estimates were used to construct the uncertainty envelopes in Figure 1. All entries refer to the draw-solution operation with the active layer facing the feed solution unless otherwise specified in the source study.

## Data Availability

The numerical data underlying all quantitative figures, the complete Figure 5 grid, observation- and system-level admissible fractions, analysis settings, and executable Python source code are provided as Supplementary Materials. The code regenerates the reported compatibility, sensitivity, and rank-diagnostic outputs from the supplied data without requiring proprietary software (version 3.13.0, Python Software Foundation, Wilmington, DE, USA). All the material detailed in the Supplementary Material is available on request to the corresponding author.

## Supplementary Materials

The following supporting information can be downloaded at: https://www.mdpi.com/article/10.3390/membranes16100339/s1, Table S1: Sensitivity of the median admissible-volume fraction to the compatibility threshold; Table S2: Harmonized observable dataset (Figure 1, Figure 2 and Figure 3); Table S3: Soft admissibility weights at representative *R*_CP_ values (Figure 3); Table S4: CP-amplified coordinate *R*_CP_ for *R*_0_ = 0.08 g L^−1^; (Figure 4); Table S5: Per-system admissible-volume fractions at *W_thr_* = 0.22 (Figure 6c); Table S6: Observable covariance eigenvalues (Figure 6a); Table S7: Explicitly constructed five-entry extended rank-diagnostic spectrum (Figure 6b).
