# Supplementary material for: Identifiability Limits of Forward-Osmosis Membrane Transport from Water and Reverse-Solute Flux Observations
Source: Membranes (Basel). 2026 Oct 13;16(10):339. doi: 10.3390/membranes16100339 (PMC13707253; doi:10.3390/membranes16100339)
Supplement: Supplementary file 1 [file membranes-16-00339-s001.zip › Supplem_Mat_v5.pdf]

Supplementary Materials

Identifiability Limits of Forward-Osmosis Membrane Transport from Water and Reverse-Solute Flux Observations

Maurizio Viviani, Nicola Bragazzi, Simonetta Papa, Luca Giacomelli, Roberto Eggenhöffner

S1. Data availability and scope of the Supplementary Material

This Supplementary Material provides the harmonized observable dataset, numerical values underlying the quantitative figures, complete mathematical definitions, analysis settings, sensitivity and convergence checks, and the accompanying executable Python scripts. Table S1 reports threshold sensitivity; Table S2 contains the 26-point observable dataset; Tables S3-S4 support Figures 3-4; Table S5 reports system-level admissible fractions; and Tables S6-S7 support Figure 6a-b. The accompanying CSV files provide the machine-readable values for Tables S1-S7 and Figures 1-5, including observation-level results and the complete 300 x 300 numerical field underlying Figure 5. Calculations were performed in Python using NumPy, pandas, and Matplotlib. Three independent experimental case studies (Kallem et al., Bai and Kruczek, He et al.) are examined separately from the harmonized dataset in Section 2.8 of the main manuscript; the relevant numerical values are reported directly in the main text and in Table 1. No additional supplementary table is provided for these cases.

S2. Extended mathematical derivations

S2.1 Algebraic origin of the rank collapse in the observable covariance matrix

The standard FO observable triplet is (J_{w}, J_{s}, R_J). In logarithmic coordinates the three observables are $x_1 = \log J_{\mathrm{w}}$, $x_2 = \log J_{\mathrm{s}}$, and $x_3 = \log R_J$. Because $R_J = J_{\mathrm{s}}/J_{\mathrm{w}}$ by definition, one has identically:

$$\log R_J = \log J_{\mathrm{s}} - \log J_{\mathrm{w}} \quad \Rightarrow \quad x_3 = x_2 - x_1 \qquad \text{(S1)}$$

For the numerical audit, R_J was recomputed as $J_{\mathrm{s}}/J_{\mathrm{w}}$ from the same flux columns before logarithmic transformation. The normalized covariance eigenvalues were 1.000, 1.39×10^{-1}, and -5.64×10^{-17}; the sign of the last value is a floating-point eigensolver artifact, and its magnitude is reported as 5.64×10^{-17}. It is numerically zero at double precision. Using the rounded R_J display column instead would introduce a small rounding residual and is therefore inappropriate for testing the exact algebraic identity.

Explicit construction of the sample covariance matrix C

Let the dataset contain N observations and define the mean-centered log-observable matrix X with rows [$\log J_{\mathrm{w,i}}$, $\log J_{\mathrm{s,i}}$, $\log(J_{\mathrm{s,i}}/J_{\mathrm{w,i}})$]. The standard sample covariance matrix is $C = X^{T}X/(N - 1)$. Because the third column of X equals the second minus the first, det(C) = 0; the resulting normalized eigenvalues are those reported in Table S6.

S2.2 Construction of the local Fisher information matrix

The reduced forward model maps the two-dimensional parameter vector $\varphi = (A, f_d)$ to the observable coordinates (J_{w}, R_J) through:

$$J_{\mathrm{w}_{\mathrm{pred}}}(A, f_{\mathrm{d}}) = \alpha \cdot A \cdot (1 - f_{\mathrm{d}}) \qquad \text{(S2a)}$$

$$R_{J_{\text{pred}}}(A, f_{\text{d}}) = \beta \cdot \frac{f_{\text{d}}}{A} \quad \text{(S2b)}$$

The dimensional scale factors are fixed, rather than fitted separately to each observation, by the median observable reference $J_{\text{w,ref}}$ = 13.25 LMH and $R_{\text{J,ref}}$ = 0.245 g L^{-1} together with (A_{ref}, $f_{\text{d,ref}}$) = (2.2, 0.06). Thus $\alpha = J_{\text{w,ref}}/[A_{\text{ref}}(1 - f_{\text{d,ref}})]$ = 6.4072 LMH and $\beta = R_{\text{J,ref}}\, A_{\text{ref}}/f_{\text{d,ref}}$ = 8.9833 g L^{-1}. For observation i, $\sigma_{\text{Jw,i}} = c_{\text{J}}\, J_{\text{w,i}}$ and $\sigma_{\text{RJ,i}} = c_{\text{R}} \max(R_{\text{J,i}}, R_{\text{floor}})$, with $c_{\text{J}} = c_{\text{R}}$ = 0.10 and R_{floor} = 0.010 g L^{-1} in the reference analysis. The floor prevents near-zero reported ratios from imposing an arbitrarily vanishing numerical width. These quantities are declared analysis settings, not fitted membrane properties or estimates of experimental standard deviation. The local Fisher matrix is the Gram matrix of the uncertainty-scaled Jacobian, so its two rows are divided by σ_{Jw} and σ_{RJ} before $J^{T}J$ is formed.

$$\mathcal{F}(A, f_{\text{d}}) = J_{\phi}^{T} J_{\phi}, \quad J_{\phi} = \frac{\partial(J_{w}^{\text{pred}}, R_{J}^{\text{pred}})}{\partial(A, f_{\text{d}})} \quad \text{(S3)}$$

The four partial derivatives of the forward map are:

$$\partial\, \partial J_{w}^{\text{pred}} / \partial A = \alpha(1 - f_{\text{d}}) \quad \text{(S4a)}$$

$$\partial J_{w}^{\text{pred}} / \partial f_{\text{d}} = -\alpha A \quad \text{(S4b)}$$

$$\partial\, \partial R_{J}^{\text{pred}} / \partial A = -\beta f_{\text{d}} / A^{2} \quad \text{(S4c)}$$

$$\partial\, \partial R_{J}^{pred} / \partial f_{\text{d}} = \beta / A \quad \text{(S4d)}$$

The eigenvalues of $\mathcal{F}(A, f_{\text{d}}) = J_{\phi}^{T} J_{\phi}$, equivalently the squared singular values of J_{ϕ}, quantify the local sensitivity of the predicted observables to displacements in the reduced parameter space. A large eigenvalue identifies a direction along which a small parameter displacement produces an appreciable change in (J_{w}, R_{J}), whereas a small eigenvalue identifies a direction along which larger parameter variations produce only limited observable changes. Because $\boldsymbol{J_{\phi}}$ maps two reduced parameters to two observables, $\boldsymbol{F}$ is a 2×2 matrix and has at most two nonzero eigenvalues. The numerical values were evaluated at the median operating point of the harmonized dataset. The relation between this reduced Fisher matrix and the five-entry diagnostic spectrum displayed in Figure 6b is described below.

Fisher spectrum in Figure 6b

Figure 6b reports an extended five-entry rank diagnostic whose construction is explicit. Entries 1–2 are the normalized eigenvalues of the 2 × 2 uncertainty-scaled local Fisher matrix evaluated at (A_{ref}, $f_{d,ref}$). Entry 3 is set to the machine-precision scale associated with the algebraic null direction in the observable covariance audit. Entries 4–5 are fixed at 10^{-20} solely as display-level structural zero-padding. Consequently, only entries 1–2 are eigenvalues of the reduced Fisher matrix; entries 3–5 must not be interpreted as additional fitted eigenvalues or parameters. The numerical vector is reported in Table S7.

S2.3 Rationale and parameterization of the logistic admissibility weights

Logistic functions were used to obtain bounded and continuously differentiable regime weights with independently specified threshold positions and transition widths. This formulation avoids discontinuities in the regime representation while retaining explicit control over the thresholds R_S and R_L and the smoothing parameters σ_S and σ_L. The unnormalized stable weight is $P_{\text{S}_{\text{un}}}(R_{\text{CP}}) = \left(1 + \exp\left(\frac{R_{\text{CP}} - R_{\text{S}}}{\sigma_{\text{S}}}\right)\right)^{-1}$.

The transition midpoint is R_{S} and the width parameter σ_{S} controls the sharpness: at $R_{\text{CP}} = R_{\text{S}} \pm \sigma_{\text{S}}$ the

weight passes through approximately 0.73 and 0.27 respectively. The same structure applies to P_L with reflected sign. After normalization to $P_S + P_T + P_L = 1$, the three weights express the relative compatibility of a given R_{CP} value with the defined transport regimes. They are not Bayesian posterior probabilities.

Numerical parameter choices

The thresholds $R_S = 0.05$ g L^{-1} and $R_L = 0.40$ g L^{-1} were selected to delimit the low-R_J, transitional, and high-R_J regions represented in the harmonized dataset. They were not estimated through an optimization procedure and should be interpreted as analysis settings rather than universal FO transport boundaries. The smoothing widths $\sigma_S = 0.012$ g L^{-1} and $\sigma_L = 0.060$ g L^{-1} were chosen to provide gradual transitions around the two thresholds. None of these four quantities is interpreted as an intrinsic membrane property. Table S3 reports the resulting normalized weights at representative R_{CP} values, allowing verification of the numerical implementation.

S2.4 Sensitivity of the admissible-volume fraction to the compatibility threshold

The admissible-volume fraction f_{adm} is the fraction of the $(A\,, f_d)$ grid satisfying $\widetilde{W} \geq W_{thr}$, where $\widetilde{W}$ is the normalized compatibility field defined by Eq. (8) of the main manuscript. The reference threshold $W_{thr} = 0.22$ was applied uniformly to all datasets. Under the diagonal Gaussian compatibility function, this threshold corresponds to a combined standardized distance of approximately 1.74 from the observed (J_w, R_J) pair.

Table S1. Sensitivity of the median admissible-volume fraction to the compatibility threshold. The median was calculated over all 26 observations using the same reduced parameter domain and numerical grid. The value $W_{thr} = 0.22$ is the reference threshold adopted in the main analysis.

W_{thr}	Median f_{adm} (all observations)
.10	1.81×10^{-2}
.15	1.49×10^{-2}
.22	1.19×10^{-2}
.30	9.45×10^{-3}
.35	8.28×10^{-3}
.40	7.19×10^{-3}

The median observation-level fraction decreased monotonically from 1.81×10^{-2} at $W_{thr} = 0.10$ to 7.19×10^{-3} at $W_{thr} = 0.40$. Width sensitivity was evaluated separately: for (c_J, c_R) = (0.05, 0.10), (0.10, 0.05), (0.10, 0.10), (0.10, 0.20), (0.20, 0.10), and (0.20, 0.20), the corresponding median fractions were 5.50×10^{-3}, 5.95×10^{-3}, 1.19×10^{-2}, 2.38×10^{-2}, 2.39×10^{-2}, and 4.79×10^{-2}. The numerical magnitude is therefore conditional on the declared resolution settings, while the existence of a finite compatibility region is not tied to a single threshold choice.

S2.5 Computational grid and convergence

The compatibility landscape $\widetilde{W}(A, f_d \mid D)$ was evaluated on a uniform domain $A \in [0.5, 4.0]$ and $f_d \in [0, 0.18]$. The reference grid contained 300 × 300 points. For each observation the field was normalized by its maximum on that same declared domain before thresholding. Observation-level f_{adm} is the number of grid nodes satisfying $\widetilde{W} \geq W_{\mathrm{thr}}$ divided by the total number of nodes. The system-level value is the median of the observation-level fractions assigned to that membrane label; single-observation systems therefore retain their individual value. Recalculation on 150 × 150 and 500 × 500 grids was used as a discretization check. The supplied script performs this check and exports the complete reference grid, avoiding dependence on graphical digitization.

S3. Numerical data underlying manuscript figures

Tables S1–**S7** provide the numerical values used to generate the figures reported in the main manuscript. Each table corresponds to the figure indicated in its title and contains the underlying numerical values rather than graphical representations.

Table S2. Harmonized observable dataset (Figures 1, 2, 3)

Complete observable transport coordinates for the membrane systems included in the analysis. Values of J_s marked with † were calculated as $J_s = R_J J_w$ because the source publication did not provide a separate tabulated value. Region labels were assigned using the analysis thresholds R_S = 0.05 g L^{-1} and R_L = 0.40 g L^{-1}.

Source	Membrane system	J_w (LMH)	J_s (g m^{-2} h^{-1})	R_J (g L^{-1})	Region
Phillip et al.	HTI-CTA	5.0	1.20 †	0.24	Transitional
Phillip et al.	HTI-CTA	9.8	2.45 †	0.25	Transitional
Phillip et al.	HTI-CTA	14.5	3.77 †	0.26	Transitional
Phillip et al.	HTI-CTA	19.8	5.15 †	0.26	Transitional
Rufuss et al.	Aquaporin HFFO.6	6.6	1.45 †	0.22	Transitional
Rufuss et al.	Aquaporin HFFO.6	7.1	1.63 †	0.23	Transitional
Rufuss et al.	Aquaporin HFFO.6	7.7	1.77 †	0.23	Transitional
Rufuss et al.	Aquaporin HFFO.6	8.1	1.78 †	0.22	Transitional
Rufuss et al.	Aquaporin HFFO.6	17.3	4.50 †	0.26	Transitional
Rufuss et al.	Aquaporin HFFO.6	17.7	4.43 †	0.25	Transitional

Source	Membrane system	J_w (LMH)	J_s (g m⁻² h⁻¹)	R_J (g L⁻¹)	Region
Rufuss et al.	Aquaporin HFFO.6	18.8	4.51 †	0.24	Transitional
Rufuss et al.	Aquaporin HFFO.6	19.6	4.51 †	0.23	Transitional
Rufuss et al.	HFFO.6 CD 1.5 M	33.0	26.40 †	0.80	Leakage-prone
Nasr et al.	PES/GO SG3	105.0	0.027	2.6×10^{-4}	Selective
Nasr et al.	PES/GO SG3	114.7	0.300	2.6×10^{-3}	Selective
Nasr et al.	PES/GO SG3	122.7	1.148	9.4×10^{-3}	Selective
Nasr et al.	PES/GO SG3	127.0	2.401	1.9×10^{-2}	Selective
Nasr et al.	HTI-CTA ref.	7.2	2.00 †	0.278	Transitional
Nasr et al.	HTI-CTA ref.	9.0	2.90 †	0.322	Transitional
Nasr et al.	HTI-CTA ref.	12.0	4.10 †	0.342	Transitional
Nasr et al.	HTI-CTA ref.	15.0	5.30 †	0.353	Transitional
Idris et al.	TFN-0GO	5.15	4.02 †	0.78	Leakage-prone
Idris et al.	TFN-0.1GO	8.50	2.38 †	0.28	Transitional
Idris et al.	TFN-0.25GO	14.65	3.66 †	0.25	Transitional
Idris et al.	TFN-0.5GO	8.50	5.02 †	0.59	Leakage-prone
Idris et al.	TFN-1.0GO	8.00	5.28 †	0.66	Leakage-prone

Table S3. Soft admissibility weights at representative R_{CP} values (Figure 3)

Normalized weights computed from Eqs. (4)–(6) of the main manuscript with R_S = 0.05 g L⁻¹, R_L= 0.40 g L⁻¹, σ_S= 0.012 g L⁻¹, σ_L = 0.060 g L⁻¹. Values at $R_{CP} = R_S$ and $R_{CP} = R_L$ confirm that each logistic function passes through 0.50 at its midpoint before normalization.

R_{CP} (g L⁻¹)	P_S (selective)	P_T (transitional)	P_L (leakage-prone)
1.0×10^{-4}	0.985	0.014	0.001
1.0×10^{-3}	0.983	0.015	0.001
1.0×10^{-2}	0.966	0.033	0.002

R_{CP} (g L⁻¹)	P_S (selective)	P_T (transitional)	P_L (leakage-prone)
5.0×10^{-2}	0.500	0.497	0.003
1.0×10^{-1}	0.015	0.978	0.007
2.0×10^{-1}	< 0.001	0.966	0.034
4.0×10^{-1}	< 0.001	0.500	0.500
8.0×10^{-1}	< 0.001	0.001	0.999

Table S4. CP-amplified coordinate R_{CP} for R_0 = 0.08 g L⁻¹ (Figure 4)

The numerical values and executable source code used for this calculation are included in the accompanying Supplementary files.

J_w (LMH)	R_{CP} (k_{eff}= 20 LMH)	R_{CP} (k_{eff} = 40 LMH)	R_{CP} (k_{eff} = 60 LMH)
0	0.0800	0.0800	0.0800
5	0.1027	0.0907	0.0870
10	0.1319	0.1027	0.0945
15	0.1694	0.1164	0.1027
20	0.2175	0.1319	0.1116
25	0.2792	0.1495	0.1214

Table S5. Per-system admissible-volume fractions at W_{thr} = 0.22 (Figure 6c)

Fractions calculated from the normalized compatibility field of Eq. (8) at W_{thr} = 0.22, using the 300×300 grid described in Section S2.5. Systems are ordered by increasing admissible-volume fraction. Threshold sensitivity is reported in Section S2.4.

Membrane system	Source	R_J range (g L⁻¹)	f_{adm} at W_{thr} = 0.22
PES/GO SG3	Nasr et al.	2.6×10^{-4}–1.9×10^{-2}	6.28×10^{-4}
TFN-0.1GO	Idris et al.	0.28	9.41×10^{-3}
TFN-0GO	Idris et al.	0.78	1.08×10^{-2}
HTI-CTA ref.	Nasr et al.	0.278–0.353	1.92×10^{-2}

Membrane system	Source	R_J range (g L^{-1})	f$_{adm}$ at W_{thr} = 0.22
HTI-CTA	Phillip et al.	0.24–0.26	1.95×10^{-2}
TFN-0.5GO	Idris et al.	0.59	2.39×10^{-2}
TFN-1.0GO	Idris et al.	0.66	2.42×10^{-2}
Aquaporin HFFO.6	Rufuss et al.	0.22–0.26	2.43×10^{-2}
TFN-0.25GO	Idris et al.	0.25	2.73×10^{-2}
HFFO.6 CD 1.5 M	Rufuss et al.	0.80	2.98×10^{-2}

Table S6. Observable covariance eigenvalues (Figure 6a)

Normalized eigenvalues of the sample covariance matrix of (log J_w, log J_s, log R_J) computed over the 26 observations of Table S2. The third eigenvalue confirms algebraic rank collapse as derived in Section S2.1.

Eigenvalue index	Normalized eigenvalue λ_k	Interpretation
1	1.000	Dominant independent observable direction
2	1.39×10^{-1}	Second independent observable direction
3	5.64×10^{-17}	Algebraically dependent direction ($\log R_J = \log J_s - \log J_w$)

Table S7. Explicitly constructed five-entry extended rank-diagnostic spectrum (Figure 6b)

Normalized values for the explicitly constructed diagnostic are reported below. No additional numerical-rank threshold is imposed.

Entry index	Normalized value	Identifiability status
1	1.000	Local Fisher direction 1
2	6.46×10^{-4}	Local Fisher direction 2
3	2.22×10^{-16}	Observable algebraic-null scale
4	1.0×10^{-20}	Constructed structural-null display entry

Entry index	Normalized value	Identifiability status
5	1.0×10^{-20}	Constructed structural-null display entry

S4. Data availability and reproducibility statement

The numerical data underlying all quantitative figures are supplied in Tables S1-S7 and in the accompanying machine-readable CSV files. Table_S1_threshold_sensitivity.csv, Table_S2_observable_data.csv, Table_S3_regime_weights.csv, Table_S4_CP_curves.csv, Table_S5_system_admissible_fractions.csv, Table_S6_covariance_eigenvalues.csv, and Table_S7_extended_diagnostic_spectrum.csv reproduce Tables S1-S7. Figure_1_R0_contours.csv, Figure_2_selected_data.csv, Figure_3_weight_curves.csv, Figure_3_experimental_positions.csv, Figure_4_curve_data.csv, and Figure_5_landscape_grid.csv contain the machine-readable data underlying Figures 1-5. observation_level_admissible_fractions.csv records the observation-level values before system aggregation, compatibility_width_sensitivity.csv reports the declared width-sensitivity analysis, and grid_convergence.csv reports the numerical grid-convergence check. The accompanying scripts Figure_1.py through Figure_6.py reproduce the six manuscript figures, while FO_reproducibility_tables.py regenerates Tables S1-S7 and the additional numerical diagnostics.

The figure-generation scripts are self-contained. FO_reproducibility_tables.py uses only the supplied Table_S2_observable_data.csv file as input. No proprietary data, external database, or proprietary software is required.

The observable dataset in Table S2 was assembled from Phillip et al. (Environmental Science & Technology, 2010), Rufuss et al. (Desalination, 2023), Nasr et al. (RSC Advances, 2022), and Idris et al. (Membranes, 2020). The three independent experimental case studies examined in Section 2.8 of the main manuscript draw on Kallem et al. (Journal of Environmental Chemical Engineering, 2022, 10, 107454), Bai and Kruczek (Membranes, 2022, 12, 385), and He et al. (Chemical Engineering Journal, 2024, 484, 149492). These three studies were not pooled with the harmonized dataset and their data values are reported in the main text. Where a source reported R_J without a separate tabulated J_s, the values marked with † were calculated as $J_s = R_J J_w$. Unit conversions were applied consistently across the combined dataset.

The regime thresholds and smoothing widths were fixed at the values reported in Section S2.3. Section S2.4 evaluates sensitivity to the compatibility threshold W_{thr}, and Section S2.5 evaluates grid convergence. These analyses establish robustness with respect to the tested threshold and grid resolution; they do not constitute a global sensitivity analysis of all model parameters.
